# William H. Atkinson

**Published:** 1891-06

**Authors:** 


DIED, at New York City, April 2, 1891, of pneumonia, WILLIAM H. ATKINSON, M. D., D. D. S., in the seventy-seventh year of his age.

Dr. Atkinson was born in Newton, Bucks Co., Pa., January 23, 1815. He graduated in medicine at the Sterling Medical College, Ohio, in 1847, and received the degree of D. D. S. from the Ohio College of Dental Surgery in 1859. He was married in 1840, at Meadville, Pa., to Martha C. Woodruff. He began the practice of medicine in Meadville in partnership with Dr. William Woodruff, his preceptor and father-in-law, and from thence removed to Norwalk, Ohio, continuing the practice of medicine and surgery. Becoming interested in dental practice, he removed to Cleveland, Ohio, and formed a partnership with Dr. Slawson. Dr. Charles R. Butler was his first dental student, with whom he subsequently formed a partnership. He removed to New York in 1861, and remained in practice there until the close of his life.

Dr. Atkinson was connected as an honorary or active member, or presiding officer, with most of the more important local dental societies; was for a short time professor of the institutes of medicine in the New York College of Dentistry; served as president of the American Dental Association, and was a frequent contributor to the periodical literature of dentistry. For many years he was a prominent figure in the ranks of his profession, bearing the title of "Father" or "Pop" Atkinson, bestowed upon him in affectionate recognition of his readiness to impart instruction to all who desired or needed it. An allusion in dental circles to "the grand old man" was always accepted as referring to him. His heartiness of manner and speech endeared him to his associates; gained the attention and won the regard of those with whom he was brought into fresh contact. He was perhaps more widely known throughout the country as a prominent dentist than any man now living,—his reputation in this respect not being confined to his fellow-practitioners but extending to the general public.

Dr. Atkinson was an excellent operator,—had indeed few equals; was skilled in all the departments of his profession, and was well versed in the pathology and thera peutics of dentistry. He was a fluent talker, an enthusiast in his calling, and desirous of arousing a like enthusiasm in others. He was a dogmatic teacher, and impressed his hearers by his positiveness into acceptance of his theories, but his style of composition was so peculiar, and so marked by a lofty transcendentalism, as to negative to a great extent his usefulness as a writer and speaker.

He was a constant attendant at all dental societies with which he was connected, and a frequent and always a welcome visitor to other dental organizations. He seldom, if ever, attended a dental meeting without being called upon to speak on one or more topics under discussion, and never failed to respond, not infrequently making his extempore contribution the feature of the occasion.

It will, we think, be generally admitted that Dr. Atkinson has done more than any other one man to establish a fraternal feeling among dentists, and to promote in dental organizations the full and free discussion of all matters of professional interest, thus markedly stimulating the professional spirit among the practitioners of dentistry.

The closing years of his life were saddened by the death of his wife and the loss within a short time of two sons who were engaged in dental practice.

He leaves one son, Dr. Charles B. Atkinson, who was associated with him in practice, and five daughters, three of whom married dentists. Another son, Dr. Wm. F.

Atkinson, died only three days previous to the death of his father.

Dr. Charles F. Allan, who had been on intimate relations with Dr. Atkinson from boyhood aud who doubtless voices the sentiment of a large number of the profession, writes as follows:

"Our dear friend was, in a broad-meaning way, a one-sided man. He loved his profession and his fellow-man, and there was no other side to him. In that 'fulfilling of the law' was the mainspring of all his energy, of everything that made toward his success in life. . . .

"Every dentist thrown into personal relations with him felt the magnetism of his profesional love. The energy of strong conviction sometimes gave a seeming asperity to his professional arguments that he was only too glad to smooth over and disavow, as from a personal stand-point, at the first opportunity.

"His residence in New York was a rallying point for the profession from all over the world, and his hospitality, unfortunately perhaps, was unbounded and never limited by his income.

His professional work and standing and its relative and actual influence with dentists is not within the scope of this brief notice, but probably all will agree that no one man has been a more important factor in the elevation of our profession.

"He was a student all his life. 'Good enough' was never good enough for him if he could do better, and his enthusiasm for good work was apt to prevail with all who were brought within his influence. . . .

"All the members of a great profession will mourn his loss as that of a dear friend and valued mentor."—*Dental Cosmos.*

